# 

**Published:** 1912-12-28

**Authors:** 


### Gifts and Bequests.

The Chelsea Hospital for Women has received for its Rebuilding Fund £105 from Messrs. Rothschild and £50 from Sir Philip Sassoon, Bart.

Sir William Treloar has received a cheque for £1,000 from "C. D. B." as a Christmas donation to the Lord Mayor Treloar Cripples' Hospital and College at Alton.

Mrs. Elizabeth Mary Laing, Barnes, has left £2,000 to the West London Hospital and £1,000 to the Huddersfield Royal Infirmary. The amount available for charitable purposes would appear to be over £40,000.

### A COLLEAGUE'S SUCCESS.

One of the oldest supporters of The Hospital, who did a great deal of strenuous work for the paper in its early days, is Mrs. Furley Smith, wife of the late Dr. Furley Smith, a much-respected medical man. It gives us, therefore, great pleasure respectfully to congratulate a former member of our staff on the success which has just attended her younger son at Oxford, where, at the age of barely eighteen, he has gained a scholarship at New College. *Quod bonum, felix, faustumque sit!*

